# Age-Related Psychophysiological Vulnerability to Phenylalanine in Phenylketonuria

**DOI:** 10.3389/fped.2014.00057

**Published:** 2014-06-23

**Authors:** Vincenzo Leuzzi, Daniela Mannarelli, Filippo Manti, Caterina Pauletti, Nicoletta Locuratolo, Carla Carducci, Claudia Carducci, Nicola Vanacore, Francesco Fattapposta

**Affiliations:** ^1^Department of Paediatrics, Child Neurology and Psychiatry, Sapienza Università di Roma, Rome, Italy; ^2^Department of Neurology and Psychiatry, Sapienza Università di Roma, Rome, Italy; ^3^Department of Experimental Medicine, Sapienza Università di Roma, Rome, Italy; ^4^Department of Molecular Medicine, Sapienza Università di Roma, Rome, Italy; ^5^Centre for Epidemiology, Surveillance and Health Promotion (CNESPS), Istituto Superiore di Sanità, Rome, Italy

**Keywords:** PKU, event-related potentials, event-related potentials in PKU, neurocognitive outcome in PKU

## Abstract

**Background:** Phenylketonuria (PKU) is caused by the inherited defect of the phenylalanine hydroxylase enzyme, which converts phenylalanine (Phe) into tyrosine (Tyr). Neonatal screening programs and early treatment have radically changed the natural history of PKU. Nevertheless, an increased risk of neurocognitive and psychiatric problems in adulthood remains a challenging aspect of the disease. In order to assess the vulnerability of complex skills to Phe, we explored: (a) the effect of a rapid increase in blood Phe levels on event-related potentials (ERP) in PKU subjects during their second decade of life; (b) the association (if existing) between psychophysiological and neurocognitive features.

**Methods:** Seventeen early-treated PKU subjects, aged 10–20, underwent ERP [mismatch negativity, auditory P300, contingent negative variation (CNV), and Intensity Dependence of Auditory Evoked Potentials] recording before and 2 h after an oral loading of Phe. Neurocognitive functioning, historical and concurrent biochemical values of blood Phe, Tyr, and Phe/Tyr ratio, were all included in the statistical analysis.

**Results:** Event-related potential components were normally detected in all the subjects. In subjects younger than 13 CNV amplitude, W2-CNV area, P3b latency, and reaction times in motor responses were negatively influenced by Phe-loading. Independently from the psychophysiological vulnerability, some neurocognitive skills were more impaired in younger patients. No correlation was found between biochemical alterations and neurocognitive and psychophysiological findings.

**Conclusion:** The vulnerability of the emerging neurocognitive functions to Phe suggests a strict metabolic control in adolescents affected by PKU and a neurodevelopmental approach in the study of neurocognitive outcome in PKU.

## Introduction

Phenylketonuria (PKU; OMIM#261600) is an inherited metabolic cause of mental disability due to the defect of the phenylalanine hydroxylase (PAH) enzyme that converts phenylalanine (Phe) into tyrosine (Tyr). Neonatal screening programs and early treatment have radically changed the natural history of PKU (1). Despite the favorable clinical outcome of early-treated PKU subjects, when compared with late- or untreated patients, an intelligence quotient (IQ) lower than expected (2) and minor neuropsychological (3) and psychiatric (4) problems remain challenging aspects of the disease. The pathogenesis of these alterations is unknown. A disorder of central dopaminergic and serotoninergic connectivity has been suggested (5). In general terms, the risk of failure in attaining the expected level of mental development in early-treated PKU patients is related the quality of the dietary control during the first years of life (6, 7). A specific vulnerability of nervous structures to Phenylalanine (Phe) in their critical period of development has been suggested by neuropathological (8) and neurophysiological studies (9). If such concept may be extended to the latest stages of high cortical function development, during adolescence and early adulthood, it is just a matter of conjectures. This is an important issue, considered the decline of dietary compliance after the first decade of life with several patients failing to comply with or abandoning the diet (10). Presently, there are no clinical or instrumental tools capable to establishing the potential age- and/or individual-related risk of neurocognitive impairment in case of interruption of the diet therapy during adolescence and young adulthood.

Within this frame, the aims of the present study were: (a) exploring the effect of rapid variations in blood Phe on psychophysiological components involved in attention and information processing in early-treated young PKU subjects during their second decade of life; (b) assessing the association (if existing) between psychophysiological vulnerability, biochemical control and neurocognitive functions.

## Materials and Methods

The experimental design scheduled two subsequent steps. The neurocognitive assessment was performed at Examination 1. Examination 2, 2 or 3 weeks later, was dedicated to the psychophysiological study (see below), which was performed before (T1) and 2 h after (T2) an oral loading of Phe (100 mg/kg). The time span between T1 and T2 recordings was based on H-MRS studies, which showed a pick of brain Phe concentration 1–4 h after an oral loading of Phe (11, 12). As the loading procedure was concerned, all patients were instructed to continue their usual dietary regime until the evening before the day of the test, which was performed in the morning after an overnight fast (T1 recording). Afterward, an orange juice drink with purified Phe was administered, and 2 h later the T2 recording was started. No other meal was allowed until the accomplishment of this second phase of the test.

Phe, Tyr levels, and Phe/Tyr ratio were assessed on dry blood spot on the day of neuropsychological assessment, and before T1 and T2 examinations.

The study was approved by the medical ethical committee and a written informed consent was obtained from patients or patients’ parents (for minors).

### Subjects

Seventeen subjects were sampled among the PKU patients in charge of the Department of Paediatrics and Child Neurology and Psychiatry in Rome, according to the following criteria: (a) genetically confirmed defect of *PAH*; (b) early diagnosis, by neonatal screening program, and treatment; (c) aged 10–20; (d) IQ ≥80; (e) availability of biochemical data covering the patient’s history from the diagnosis up to the study; (f) absence of medical condition, other than PKU, affecting neuropsychological and/or psychophysiological functions; (g) no history or clinical evidence of consumption of drugs or medicaments interfering with neurocognitive and/or psychophysiological functions; and (h) no pregnancy.

Demographic and clinical characteristics of the sample are shown in Table 1.

**Table 1:** **Demographic and clinical characteristics of the PKU sample**.

Patient ID	Sex	Genotype		Biochemical phenotype	Age at diet onset (days)	Age at the study (yrs/mo)	IDC (μM) birth to 10 years and 11 months	IDC (μM) 11 years to the study	Time-gap since diet continuation (yrs/mo)
		Allele 1	Allele 2	
01	F	p.Arg261Gln	c.1066-11G > A	CPKU	14	10/1	434	–	On diet^a^
02	F	p.Arg408Trp	p.Arg408Trp	CPKU	10	10/8	486.5	–	On diet
03	M	p.Tyr414Cys	c.1066-11G > A	MPKU	37	10/9	353	–	On diet^a^
04	M	p.Leu48Ser	p.Leu369Leu	MPKU	22	10/11	356.5	–	On diet^a^
05	M	p.Asp59Val	p.Arg261Gln	MPKU	31	10/11	313	–	On diet^a^
06	F	p.Arg261Gln	p.Arg176^a^	MPKU	30	12/11	375	865	On diet
07	M	p.Tyr414Cys	p.Arg408Gln	HPA	21	13/10	346	393	3/0
08	F	p.Leu48Ser	p.Leu48Ser	MPKU	44	14/9	298	422	3/3
09	M	c.1066-11G > A	p.Arg176^a^	CPKU	15	16/3	358.5	743.5	On diet
10	M	c.1066-11G > A	p.Gly352Cys	MPKU	30	16/11	370	571^a^	3/0
11	M	p.Asp338Tyr	p.Pro281Leu	MPKU	32	17/1	322	545	3/6^a^
12	F	p.Leu194Pro	p.Arg261^a^	CPKU	16	17/3	388	1050.5	4/3
13	M	c.1066-11G > A	p.Tyr414Cys	MPKU	14	17/3	542	606	On diet^a^
14	M	p.Arg261Gln	p.Arg408Gln	MPKU	12	17/11	231	478	On diet
15	M	p.Arg408Trp	p.Arg408Trp	CPKU	25	17/11	545	754	On diet
16	F	p.Arg261Gln	p.Arg158Gln	MPKU	20	18/2	235	699	On diet
17	M	p.Pro281Leu	p.Arg158Gln	CPKU	30	20/0	600	834.75	0/5
Mean					23.7		385.5	647.9	
SD					9.69		105	198	
Range					10–44		231–600	393–1050	

*CE, clinical examination; IDC, index of dietary control, median value of Phe for the considered period; yrs/mo, years/months; CPKU, classical PKU (blood Phe at diagnosis or under free diet persistently >1200 μM); MPKU, mild PKU (blood Phe at diagnosis or under free diet persistently >600 <1200 μM); HPA, persistent hyperphenylalaninemia (blood Phe at diagnosis or under free diet persistently >360 <600 μM)*.

^a^ Under sapropterin treatment

### Neurocognitive assessment

The Neurocognitive assessment was focused on the following functions: (a) IQ [Wechsler intelligence scale for children-III (WISC-III) for patients aged 6–16 and Wechsler adult intelligence scale-revised (WAIS-R) for patients ≥17 years]; (b) cognitive flexibility and rule shifting [Wisconsin card sorting test (WCST)]. The following domains of WCST were measured: perseverative responses (PR), perseverative errors (PE), non-perseverative errors (NE), and total number of errors (*E*); (c) visuospatial and visual memory functions (Rey–Osterrieth Complex Figure Test). To the aim of the present study the accuracy scores for the copying and immediate recall were analyzed; (d) sustained and shared attention, memory functions (Leiter-R test attention and memory battery). These Leiter subtests contribute to five composite scores: memory screener, recognition memory, memory span, attention composite, and memory process.

### Biochemical studies

Phe, Tyr, and Phe/Tyr ratio were analyzed on dry blood spot by tandem mass spectrometry at each time scheduled by the protocol.

### Event-related potentials

Psychophysiological study included the following event-related potentials (ERPs): mismatch negativity (MMN); auditory P300 (oddball and with a discriminant motor response); contingent negative variation (CNV); and intensity dependence of auditory evoked potentials (IDAP). The brain functions potentially probed by the ERPs and the related behavioral tasks are reported in Table 2 (13–25).

**Table 2:** **Event-related potentials paradigm: main psychophysiological and neurochemical significance**.

Paradigm	Description	Significance	
		Psychophysiological	Neurochemical
Multi-deviant mismatch negativity (MMN)	Three consecutive blocks of acoustic stimuli were delivered binaurally via earphones. Each block consisted of a sequence of rapidly repeated standard acoustic stimuli occasionally interrupted by a rare deviant sound (a different one for each block) with a probability of 0.1. All the stimuli consisted of sinus tones (rise-fall times: 5 ms): the standard tone was 1000 Hz (duration: 150 ms; intensity: 80 dB-SPL); deviant 1 was different for its frequency (1150 Hz), deviant 2 differed from standard tone for duration (100 ms), deviant 3 differed for intensity (90 dB). The inter-stimulus interval (ISI) was fixed on 500 ms. Each block, comprising 500 stimuli, lasted about 10 min. The subjects, who were required to watch a silent movie during the task, were unaware of the occurrence of the different tones	Pre-attentive discrimination; sensory–memory updating; and change/rule violation detection (13)	NMDA receptor (14, 15); dopamine (16, 17); serotonin (18)
P300 auditory two-stimuli (oddball and motor response)	Auditory stimuli, consisting of pure tones of 200 ms duration (rise-fall times: 0 ms) and intensity of 80 dB-SPL delivered binaurally via earphones, were administered in two consecutive blocks during the same session. During each block, which consists of 150 trials, 2 tones were administered: standard stimuli (1000 Hz) and target stimuli (2000 Hz) that occurring randomly with a probability of 0.33. During the first block, the subjects were instructed to recognize the target stimuli by mental counting; during the second block they had to push a button as quickly as possible upon hearing the target tones. Inter-trial interval varied randomly between 3 and 6 s	Selective attentional processing; memory storage (19)	Norepinephrine (20); dopamine (21)
Contingent negativity variation (CNV)	It is evoked whenever a close temporal relationship between two-stimuli, i.e., “warning” and “imperative,” is established. The warning and imperative stimuli consisted, respectively, of a light flash of 100 μs–1.5 J (S1) (delivered by a strobe lamp at a distance of 30 cm from the subject) and, 1750 ms later, a sound at an intensity of 80 dB-SPL, lasting 200 ms, randomly presented at either 1000 or 2000 Hz (S2). Subjects were instructed to push a button with the right index finger as quickly as possible upon hearing the 2000-Hz sound. The inter-trial interval varied randomly between 5 and 10 s. A total of 100 trials were acquired	Alertness response; sustained attention during an operative conditioning (22)	Norepinephrine (23); dopamine (24)
Intensity dependence of auditory evoked potentials (IDAP)	Auditory evoked potentials were evoked by an acoustic stimulation consisted of four runs of 250 stimuli each with the inter-stimulus interval being randomized between 500 and 900 ms. Tones of 1000 Hz and 50 ms duration (rise and fall times: 10 ms) were delivered binaurally through earphones at four different intensities (60, 70, 80, and 90 dB) in a pseudo-randomized order. The sounds were presented and controlled by a PC running system. The subjects were not informed about the sequence of different tones and were instructed to ignore themselves. For each intensity level, at least 150 trials were collected	Serotoninergic central tone (25)	Serotonin (25)

### EEG recording

Participants were seated on an anatomic chair in a faradized and light-attenuated room. The electrophysiological signals were recorded by Ag/AgCl electrodes fixed on the scalp at the F3, Fz, F4, C3, Cz, C4, P3, Pz, and P4 sites, according to the International 10–20 System, referred to linked mastoids and grounded at Fpz. The bipolar electrooculogram (EOG) was recorded from above and below the left eye. All inter-electrode impedances were kept below 3 KΩ. EEG signals and EOG were filtered using a 0.01–30 Hz bandpass (a narrow bandpass at 2–10 Hz was applied for MMN analysis). A notch filter was also applied. The data were entered via an analog/digital converter at a sampling rate of 1024 Hz and stored in a hard disk. A Mizar Sirius EEG-EP multifunctional system was used.

### ERP analysis

All auditory signals were visually inspected and revised by one investigator (F.F.) blinded to the patient’s age and clinical data.

Trials containing eye movements were automatically rejected. A further selection was performed in the offline analysis in order to reject other kinds of artifacts, according to the clinical guidelines (26).

With regard to MMN, ERPs were obtained by averaging EEG epochs separately for all deviant and standard tones. Difference waveforms were calculated by subtracting the ERPs of standard stimuli from those elicited by each deviant stimulus. The MMN latency and amplitude for each deviant stimulus were measured from the most negative peak detected between 100 and 250 ms post the onset of the stimulus difference, at Fz, on the difference-wave.

With regard to the P3 paradigm, in the target response, the N1 component was identified as the most negative peak between 75 and 140 ms, while the P3b components were defined as the largest positive deflections between 250 and 500 ms on the stimulus onset. The amplitude of these components was identified by means of baseline-to-peak measurements. For the sessions, counting responses and mean reaction times (RTs) of correct responses were calculated (range for correct responses 100–1000 ms).

The CNV amplitude was measured as the total area (negative shift between S1 and S2) and as two temporal windows of interest: the early orienting window – W1 (between 500 and 700 ms after S1), and the late window – W2 (200 ms preceding S2) (27). The RT was evaluated as the correct motor response at S2.

With regard to IDAP, the epoch analysis for each AEP was of 600 ms with a 100 ms pre-stimulus baseline. Amplitudes of the N1 (between 50 and 150 ms post-stimulus) and P2 (between 90 and 230 ms post-stimulus) peaks were measured. The IDAP was calculated as the linear amplitude/stimulus intensity function (ASF) slope for block averages (mV/10 dB).

All ERP recorded at T1 and T2 were compared with those obtained in healthy age matched internal controls in our lab. Moreover, as the T2 recording was concerned, each subject acted as control of himself.

### Statistical analysis

The statistical analyses have been performed using *t*-test for continuous variables (unpaired and paired samples) and an exact Fisher test for categorical variables. A correlation analysis between demographic, clinical, biochemical, and neuropsychological and neurophysiological variables was also performed by means of Pearson’s index.

In order to explore the possible influence of the age on neurocognitive and psychophysiological performances, the sample was sub-grouped in tertiles according to the patients’ age and each subgroup was compared with each other with respect to neuropsychological and neurocognitive performances. All data were analyzed with SPSS (version 21.0). A *p* value ≤0.05 was considered to indicate statistical significance.

## Results

### Neurocognitive assessment

Neurocognitive assessment was accomplished in all subjects (Table 3). The mean concurrent value of Phe value in blood was 697.13 μM (±279.36). In the whole sample IQ ranged 80–124 (mean 95.06; SD 15). Among the tests exploring EFs, the scores at WSCT in six subjects were under reference value of one SD in one or more domains. With regard to attention and memory, 11 out of 17 patients obtained a score lower than normal at Leiter-R battery for attention and memory. On Rey–Osterrieth Complex Figure (Copy) Test (RCFC) and Rey–Osterrieth Complex Figure (Memory) RCFM Test the mean score of PKU patients was 35.58 (SD 21.11) and 23.82 (SD 19.96) percentile of the reference population and 10 and 11 subjects out of 17, respectively, performed ≤25th percentile.

**Table 3:** **Results of neurocognitive tests and concomitant biochemical values**.

Patient ID	IQ	Leiter-R battery attention and memory					RFTC (pc)	RFTM (pc)	Wisconsin card sorting test				Blood Phe (μM)	Blood Tyr (μM)	Blood Phe/Tyr
		MS	AM	MSp	Att	MP			PR	E	PE	NE	
01	84	**65**	**62**	**84**	91	77	10	5	92	**81**	92	92	548	35.35	15.50
02	84	93	107	93	**84**	90	10	10	100	92	100	92	651	77	8.45
03	94	**81**	**83**	**82**	**81**	87	10	5	108	**81**	108	**62**	324.5	59.7	5.4
04	103	109	104	84	75	95	60	25	108	108	108	100	580.5	50.05	11.59
05	88	**78**	97	**71**	94	92	40	30	92	92	92	100	410	76	5.39
06	83	93	94	**84**	**81**	87	10	5	**81**	**81**	**81**	92	985.5	55.45	17.77
07	84	**65**	**68**	**77**	**84**	**82**	100	30	108	100	108	92	523	70.25	7.44
08	82	**84**	92	86	87	102	50	25	**81**	**81**	**81**	87	457	42.65	10.71
09	120	100	122	93	91	104	10	70	92	98	100	100	688	33.01	20.84
10	82	**71**	**71**	**77**	91	80	60	40	**81**	88	**81**	110	552	62	8.9
11	116	115	100	128	108	130	60	20	119	119	119	124	987.72	46.13	21.3
12	81	**81**	94	**77**	**75**	**77**	25	5	90	100	92	110	1400	27.85	50.26
13	124	**81**	86	100	**84**	111	10	5	108	118	110	120	679	55	12.34
14	114	**84**	92	95	102	109	20	15	119	122	119	138	479	62.2	7.7
15	94	**74**	100	**71**	**71**	**68**	100	50	85	**76**	**81**	**62**	845.5	55.55	15.22
16	103	100	119	89	91	95	20	55	108	108	108	119	660	61.2	10.78
17	80	**56**	**50**	**73**	**75**	**61**	10	10	100	100	100	98	1081	60	18.01
M	95.05	84.11	90.64	86.11	86.17	91	35.58	23.82	98.35	96.76	98.82	99.82	697.16	54.67	14.56
SD	15.23	16	19.24	13.77	9.85	17	31.11	19.96	12.73	14.6	12.96	19.83	279.36	14.15	10.49

*M, media; SD, standard deviation; CE, clinical examination; IQ, intelligence quotient; Leiter-R MS, memory screener; Leiter-R AM, associative memory; Leiter-R MSp, memory span; Leiter-R Att, attention; Leiter-R MP, memory process; RFTC, Rey figure test with copy (percentile of reference population); RFTM, Rey figure test from memory (percentile of reference population); WCST, Wisconsin card sorting test; WCST PR, WCST perseverative responses; WCST E, WCST errors; WCST PE, WCST perseverative errors; WCST NE, WCST non-perseverative errors. Less than normal values in the bold font*.

Intelligence quotient was correlated with: WCST PR (*r* = 0.595; *p* = 0.012), WCST E (*r* = 0.687; *p* = 0.002), WCST PE (*r* = 0.658; *p* = 0.004), and WCST NE (*r* = 0.480; *p* = 0.051). IQ and EFs were not significantly different in subjects on and out diet when the study was performed and neither of them was correlated with subjects’ age, and historical and/or concurrent biochemical alterations.

### Psychophysiological assessment

Mean blood Phe concentration at T1 and T2 was 571.64 μM (±192.92) and 1129 μM (±177.31), respectively (*p* < 0.001).

**Figure 1:**
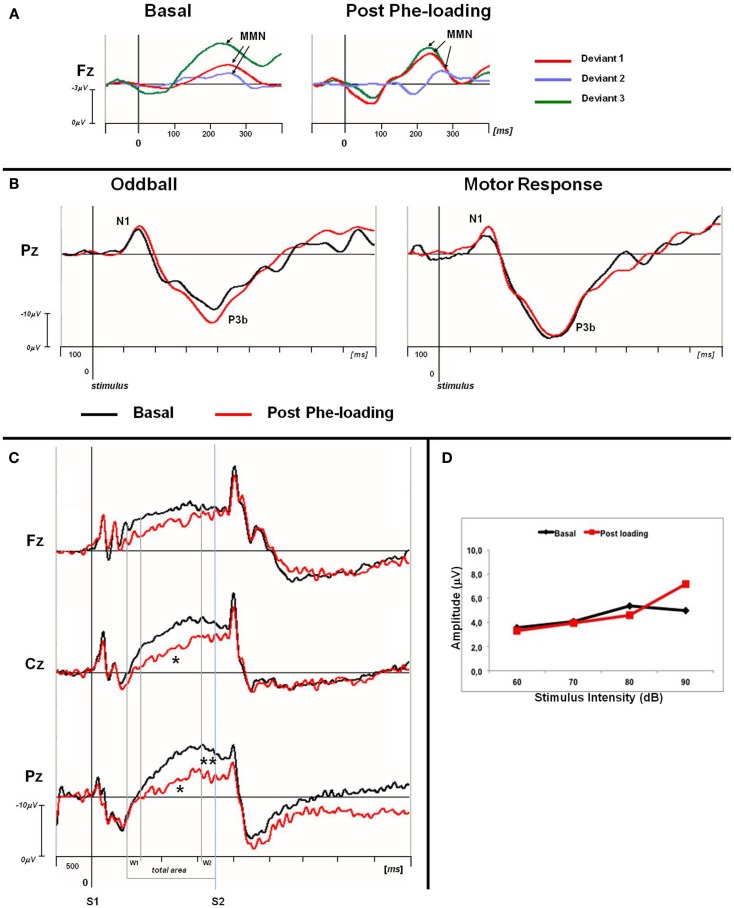
**(A)** Grand averaged MMN traces at Fz for basal and post Phe-loading condition. Difference waves are showed for each deviant stimulus (deviant 1: red line; deviant 2: blue violet line; deviant 3: green line). The analysis epoch for the MMN is 500 ms with a 100 ms pre-stimulus baseline. The arrows indicate the MMN peak for each difference waves. **(B)** Grand averaged P3 traces for oddball and motor response task at Pz site. For each task the basal (black) and post-loading trace (red) are superimposed. The epoch analysis of the P3 components is 1 s with a 100 ms pre-stimulus baseline. **(C)** Grand averaged CNV waveforms, with W1, W2, and total areas highlighted, superimposed at midline scalp sites for basal (black lines) and post-loading (red lines) evaluation. The epoch analysis is 5 s, with a 500 ms pre-stimulus baseline before S1. S1: warning stimulus (flash). S2: imperative stimulus (tone). The asterisks indicate statistical significant differences in CNV parameters (total area *p < 0.05; W2-CNV **p < 0.05). **(D)** ASF slopes of PKU subjects in basal (black) and post Phe-loading (red) condition.

Normal IDAP values and MMN waves morphology were obtained from all the participants, either in the basal condition or post Phe-loading (Figure 1; Table 4). No variations of amplitude and/or latency of MMN could be detected after the Phe-loading. No correlations were found between IDAP or MMN parameters and clinical, biochemical, and neuropsychological data.

**Table 4:** **Means and standard deviations of biochemical parameters and ERPs values in PKU subjects**.

		T1 (*n* = 17)	T2 (*n* = 17)	p*	≤13 years (*n* = 6)			>13 years (*n* = 11)		
					T1	T2	p*	T1	T2	p*
Blood Phe (μM)		571.6 ± 192.9	1129.2 ± 177.3	***<*0.001**	457.3 ± 200.5	1120.8 ± 253.7	*****<***0.001**	634.4 ± 165.3	1133.5 ± 134.4	*****<***0.001**
Blood Tyr (μM)		58.9 ± 35.2	63.6 ± 37.9	0.70	52.6 ± 16.9	62.7 ± 39.4	0.58	62.3 ± 42.5	64.2 ± 39.1	0.91
Blood Phe/Tyr		12.2 ± 8.2	21.9 ± 8.2	**0.001**	9.8 ± 5.2	21.7 ± 8.7	**0.02**	13.4 ± 9.2	22.8 ± 8.5	**0.04**
*IDAP*	Cz	0.6 ± 0.9	1.2 ± 1.9	0.22	0.8 ± 1.3	2.3 ± 3.1	0.17	0.5 ± 0.6	0.6 ± 0.6	0.55
**MMN**										
Deviant 1
*Lat* (ms)	Fz	261.1 ± 44.8	239.8 ± 47.9	0.33	230.6 ± 28.7	247.1 ± 44.5	0.41	263.3 ± 37.9	240.9 ± 51.6	0.18
*Amp* (μV)	Fz	-2.1 ± 1.8	-2.9 ± 1.5	0.23	-3.6 ± 0.7	-3.5 ± 1.5	0.79	-1.4 ± 1.7	-2.5 ± 1.5	0.11
Deviant 2
*Lat* (ms)	Fz	250.4 ± 37.4	273.8 ± 28.6	0.18	228.9 ± 65.2	279.2 ± 30.1	0.30	250.5 ± 24.3	271.6 ± 29.3	0.13
*Amp* (μV)	Fz	-1.2 ± 2.2	-1.3 ± 1.5	0.27	-1.9 ± 3.2	-1.2 ± 2.6	0.51	-0.3 ± 1.8	-1.3 ± 1.1	0.13
Deviant 3
*Lat* (ms)	Fz	237.2 ± 35.4	239.3 ± 27.5	0.93	246.3 ± 40.7	250.5 ± 36.8	0.69	234.8 ± 33.8	232.5 ± 19.3	0.80
*Amp* (μV)	Fz	-3.9 ± 1.9	-3.1 ± 2.5	0.16	-5.2 ± 1.3	-3.9 ± 2.5	0.31	-3.3 ± 2.1	-2.6 ± 2.5	0.47
**P300**										
Counting										
*Lat* (ms)
N1	Fz	148.3 ± 24.5	128.6 ± 22.8	0.07	170.7 ± 15.5	132.5 ± 29.5	0.06	136.1 ± 19.4	126.4 ± 19.9	0.27
	Cz	131.2 ± 17.2	127.1 ± 18.8	0.46	144.1 ± 17.8	136.6 ± 25.4	0.44	124.2 ± 12.7	121.9 ± 12.6	0.62
	Pz	126.6 ± 25.4	131.1 ± 21.1	0.92	137.1 ± 32.5	135.6 ± 18.9	0.91	120.8 ± 19.9	128.6 ± 22.6	0.37
P3b	Fz	381.9 ± 33.9	395.3 ± 40.8	0.13	389.5 ± 31.4	408.0 ± 53.1	0.12	378.7 ± 37.8	381.7 ± 25.8	0.81
	Cz	377.2 ± 28.4	402.3 ± 63.1	0.11	391.8 ± 29.8	428.8 ± 87.7	0.21	368.5 ± 26.7	380.4 ± 30.4	0.33
	Pz	398.4 ± 34.1	392.5 ± 60.7	0.59	410.9 ± 38.1	425.8 ± 86.1	0.42	371.9 ± 27.7	377.8 ± 27.5	0.57
*Amp* (μV)
N1	Fz	−11.2 ± 7.2	−11.6 ± 7.6	0.81	−16.5 ± 9.6	−17.3 ± 9.8	0.75	−8.3 ± 3.3	−8.5 ± 3.9	0.84
	Cz	−8.5 ± 6.5	−9.3 ± 6.1	0.55	−11.8 ± 9.4	−13.1 ± 7.6	0.69	−6.6 ± 3.4	−7.2 ± 3.8	0.62
	Pz	−7.1 ± 5.6	−6.8 ± 6.2	0.87	−10.5 ± 7.7	−9.3 ± 8.3	0.81	−5.2 ± 3.3	−5.4 ± 4.5	0.90
P3b	Fz	2.3 ± 9.4	3.4 ± 8.6	0.91	0.6 ± 10.4	3.1 ± 12.1	0.54	4.4 ± 8.6	3.6 ± 6.4	0.61
	Cz	9.7 ± 11.5	11.6 ± 7.8	0.76	9.5 ± 14.7	12.3 ± 8.6	0.65	11.4 ± 9.1	11.3 ± 7.6	0.91
	Pz	16.6 ± 12.6	18.5 ± 8.6	0.91	13.3 ± 18.2	18.4 ± 8.8	0.54	19.6 ± 8.4	18.5 ± 8.9	0.63
Motor response *Lat* (ms)										
N1	Fz	138.8 ± 21.6	149.8 ± 34.1	0.30	148.6 ± 23.9	168.3 ± 30.6	0.28	134.4 ± 20.2	138.7 ± 32.9	0.49
	Cz	131.5 ± 19.9	144.5 ± 31.1	0.39	123.6 ± 27.9	164.8 ± 29.3	0.15	135.1 ± 15.5	135.9 ± 30.2	0.89
	Pz	125.9 ± 26.1	133.4 ± 20.8	0.75	119.9 ± 30.9	149.4 ± 15.7	0.14	128.6 ± 24.8	127.1 ± 20.2	0.86
P3b	Fz	371.9 ± 37.3	387.2 ± 37.1	0.18	377.1 ± 24.9	415.7 ± 60.6	0.36	363.1 ± 36.3	372.8 ± 13.7	0.34
	Cz	370.9 ± 45.2	377.5 ± 28.6	0.37	397.6 ± 34.6	398.6 ± 23.0	0.97	350.0 ± 37.1	365.2 ± 23.6	0.22
	Pz	373.9 ± 43.5	381.1 ± 48.1	0.67	393.3 ± 37.7	414.8 ± 61.7	0.59	365.1 ± 44.7	363.1 ± 34.1	0.84
*Amp* (μV)
N1	Fz	−8.7 ± 6.3	−13.5 ± 10.1	0.16	−10.2 ± 8.9	−19.9 ± 8.6	0.09	−8.1 ± 5.0	−8.3 ± 5.1	0.88
	Cz	−7.9 ± 6.1	−9.8 ± 6.9	0.95	−9.3 ± 9.1	−16.1 ± 6.7	0.19	−7.2 ± 4.6	−6.1 ± 3.8	0.33
	Pz	−5.7 ± 4.3	−7.1 ± 7.7	0.87	−7.7 ± 6.4	−12.7 ± 8.1	0.36	−4.7 ± 2.8	−3.2 ± 3.9	0.28
P3b	Fz	3.1 ± 6.9	4.6 ± 7.3	0.91	1.7 ± 6.2	1.5 ± 10.4	0.96	4.8 ± 7.4	5.8 ± 6.2	0.44
	Cz	10.6 ± 8.8	9.6 ± 7.4	0.55	12.9 ± 9.7	6.5 ± 5.2	0.12	11.0 ± 8.1	12.3 ± 6.4	0.41
	Pz	21.4 ± 8.7	21.1 ± 8.2	0.75	25.7 ± 10.2	21.2 ± 8.3	0.34	19.9 ± 7.9	22.1 ± 8.2	0.14
RT (ms)		423.3 ± 116.8	443.7 ± 139.6	0.17	551.3 ± 64.9	607.3 ± 82.7	**0.05**	553.4 ± 67.4	554.5 ± 54.3	0.96
**CNV**										
Tot area (μV)	Fz	−8441.2 ± 4370.5	−6376.5 ± 5976.2	0.06	−8016.7 ± 4675.5	−3633.4 ± 3245.7	**0.03**	−8672.7 ± 4411.5	−7872.7 ± 6700.6	0.52
	Cz	−8558.2 ± 4488.8	−5600.0 ± 5041.2	**0.02**	−9433.9 ± 5795.4	−2816.6 ± 4159.8	**0.02**	−8081.8 ± 3838.9	−7118.2 ± 4982.7	0.36
	Pz	−7194.1 ± 5184.7	−3705.6 ± 4033.8	**0.02**	−10433.0 ± 7085.5	−1900.0 ± 2345.2	**0.03**	−5427.2 ± 2860.4	−4690.5 ± 4501.4	0.43
W1	Fz	−960.7 ± 901.7	−643.3 ± 1290.4	0.13	−704.1 ± 739.4	−378.2 ± 777.3	0.32	−1100.6 ± 983.4	−787.9 ± 1515.4	0.19
	Cz	−429.3 ± 688.5	−282.9 ± 956.0	0.26	−29.2 ± 390.8	78.3 ± 337.6	0.67	−648.1 ± 730.4	−479.9 ± 1133.2	0.37
	Pz	31.6 ± 869.5	−132.0 ± 1006.4	0.71	655.0 ± 684.3	324.4 ± 465.6	0.42	−308.7 ± 784.4	−381.1 ± 1148.4	0.84
W2	Fz	−1508.7 ± 810.4	−1263.6 ± 1230.9	0.29	−1358.3 ± 955.4	−635.1 ± 1124.8	0.11	−1591.1 ± 757.2	−1606.4 ± 1193.9	0.95
	Cz	−1786.3 ± 1047.7	−1390.6 ± 1287.8	0.28	−2068.7 ± 1445.1	−734.9 ± 1409.4	**0.05**	−1632.5 ± 798.8	−1748.3 ± 1123.2	0.61
	Pz	−1662.1 ± 1208.2	−901.8 ± 910.7	**0.05**	−2498.5 ± 1609.5	−426.1 ± 848.8	**0.006**	−1205.9 ± 625.8	−1161.3 ± 869.9	0.79
RT (ms)		337.9 ± 88.8	363.4 ± 115.3	0.14	439.2 ± 62.8	485.3 ± 90.2	0.08	282.7 ± 33.9	296.9 ± 58.6	0.38

* t-test.

*Significant data are in the bold font*.

The P3 components were well represented in all the subjects. No differences were found in the N1 and P3b (counting and motor response) amplitudes and in latencies between T1 and T2 recordings. Moreover, the number of correct responses and RTs were comparable in both the electrophysiological recordings. No correlations emerged between the P3 parameters and other clinical, biochemical, and neuropsychological data, except for a negative correlation between age and P3b latency either in counting or in motor response in T2 recording session (counting P3 latency: Fz: *r* = −0.512, *p* = 0.043; Cz: *r* = −0.486, *p* = 0.057; Pz: *r* = −0.433, *p* = 0.09; motor response P3 latency: Fz: *r* = −0.541, *p* = 0.046; Cz: *r* = −0.568, *p* = 0.027; Pz: *r* = −0.379, *p* = 0.137). A further marginal negative correlation emerged between IQ and P3 latencies at T2 (counting: Pz: *r* = −0.484, *p* = 0.057; motor response: Pz: *r* = −0.382, *p* = 0.131).

Contingent negative variation waves were elicited with a well detectable shape (28) in all the subjects; the total CNV area (Fz: *p* = 0.06; Cz: *p* = 0.02; Pz: *p* = 0.02), as well as the W2-CNV area at the Pz site (*p* = 0.05) were smaller at T2 than T1. RT values were comparable between the two ERP recording sessions.

### Subgroup analysis

**Figure 2:**
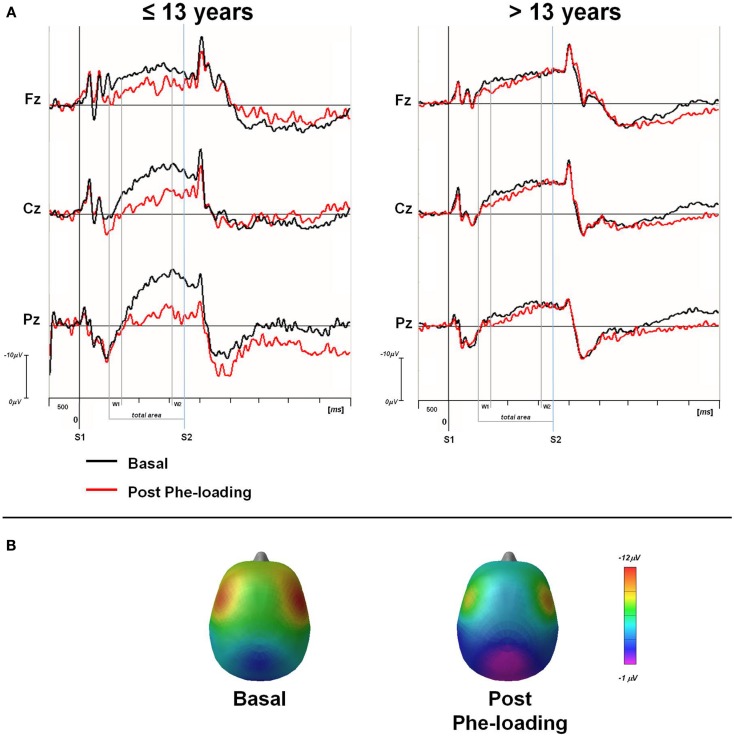
**(A)** Grand averaged CNV waveforms, with W1, W2, and total areas highlighted, superimposed at midline scalp sites for the two groups of PKU subjects (≤13 and >13 years). CNV was obtained in basal (black line) and post Phe-loading (red line) condition. S1: warning stimulus (flash). S2: imperative stimulus (tone). **(B)** Scalp potential maps at 1650 ms (mean value of W2-CNV) for ≤13 years PKU subjects in basal and post Phe-loading condition.

Once the sample was sub-grouped in tertiles according to the age of the patients, only those younger than 13 showed a significant delay in RTs in P3 motor responses after the Phe-loading respect to basal evaluation (*p* = 0.05). Moreover in the same patients, the total CNV (Fz: *p* = 0.03; Cz: *p* = 0.02; Pz: *p* = 0.03) and the W2-CNV areas (Cz: *p* = 0.05; Pz: *p* = 0.006) were smaller after Phe-loading than in basal condition (Figure 2). None of such alterations was detected in subjects older than 13 years under the effect of Phe-loading. The blood Phe concentration (micromolar) at the T2 test was not significantly different in patients younger and older than 13 (1120.8 ± 253.7 vs. 1133.5 ± 134.4).

Re-examining neurocognitive results in the light of the psychophysiological data, patients younger than 13 were more impaired in the following tests: WCST total number of errors (*p* = 0.036); RCFC trial (*p* = 0.04) and immediate recall trial (*p* = 0.03); Leiter-R attention sustained subtest (*p* = 0.027); and spatial memory subtest (*p* = 0.039). They had also a relatively lower IQ [89.33 (SD 7.84) vs. 98.19 (SD 17.61); *p* = 0.132]. Moreover, a positive correlation between IQ and RCFC (*r* = 0.759; *p* < 0.01) was observed only in this group. The differences among subgroups (younger and older than 13) could not be explained on the base of any of demographic and treatment variables showed in Table 1.

## Discussion

While we found a normal pattern of ERPs in early-treated PKU patients during their second decade of life, a selective psychophysiological vulnerability to a rapid increase of blood Phe could be demonstrated only in PKU subjects younger than 13. A mild neurocognitive impairment was also found in these patients with respect to the older subjects.

Mismatch negativity latency and amplitudes were preserved in early-treated PKU patients whatever the concomitant and historical quality of biochemical control. We cannot confirm the correlation between lifetime and concurrent Phe level and MMN amplitudes (29). However, we cannot rule out an age-related vulnerability of MMN (30), since our patients were older than those studied by de Sonneville et al. (29). In addition, our result shows that an acute increase in Phe does not affect the pre-attentive auditory discrimination processes, as demonstrated by the short term stability of MMN parameters after Phe-loading. We can therefore hypothesize that the connectivity between the auditory and the frontal cortices, that is thought to be is crucial for the genesis of the MMN phenomenon (13), is preserved in early-treated PKU subjects during their second decade of life.

In our subjects, IDAP value was not affected by Phe-loading. This is an interesting result since IDAP is thought to be inversely related to central serotonin neurotransmission in humans and in animal models (31), and serotonin metabolism and transmission are potentially affected by the increase of Phe levels in blood and brain (32, 33).

P300 is produced when one attends to a stimulus, and it is often interpreted as the first major component reflecting selective attentional processing of the stimulus (34).

Observing that P300 latency is influenced by levodopa treatment in Parkinson disease (21) has suggested it as possible component of dopaminergic network. Two relevant results emerged from our study as far as P300 is concerned: (a) an inverse correlation between age and P3b latency after Phe-loading; (b) a significant delay in RTs in motor responses in younger patients after Phe-loading. Both data suggest a specific vulnerability of the network generating this potential to Phe according to patients’ age. A severe derangement of P300 wave under oddball auditory paradigm was found in late-treated PKU patients, while early-treated patients showed an increase of P300 latency and amplitude unrelated to historical and/or concomitant levels of Phe (35). These findings were not confirmed in younger early and continuously treated PKU patients, which were studied with a visual discriminative paradigm (36). In another study, de Sonneville et al. (37) confirmed the absence of differences for selection positivity over anterior sites (P180) associated with target detection in children with PKU. Moreover, negative (N90, N180) and positive (P180) selection potentials over fronto-central sites seemed to be depending on concurrent and historical Phe levels, whereas sensory potential (N170) depended more strongly on historical Phe levels. They also found a higher number of false positive responses on orientation relevant and irrelevant trial in PKU as compared with controls, and less accurateness on PKU subjects with higher value of lifetime Phe levels in blood. Both studies confirm a correlation between age and RT. In our study, we are not able to confirm such correlation.

Contingent negative variation is associated with activation in the prefrontal cortex (PFC) (38, 39) potentially one of the most vulnerable brain structure in PKU patients because of the peculiar fragility of dopaminergic connectivity in this region (40, 41). The cognitive generator is simple attentional anticipation: a first warning stimulus is a cue that a second stimulus is about to appear, to which the subject responds (usually by pressing a key). When the initial response to the warning stimulus is completed, a negativity develops up until the second stimulus appears. It has been shown that such negativity is correlated to the performance on psychometric measures associated with executive functions, and by extension to PFC functioning (42–44). The increase of CNV amplitudes with the age over the vertex has suggested a maturational trend of this potential (30, 42, 45).

In all subjects, we recorded a normal pattern of the CNV waves in basal condition. However, a couple of hour after Phe-loading, a derangement of the total CNV amplitude emerged suggesting a suppression of the cerebral on-going activity thought to prepare the attentional system for a rapid motor response after a warning signal (24). Such alerting response seems to be compromised particularly in its late phase (W2-CNV), thus indicating a peculiar dysfunction of the premotor cerebral activation for a motor response. Conversely, the orienting phase of this attentional reaction is preserved, as suggested by the stability of the early orienting CNV window (W1-CNV). Interestingly, such dysfunction in CNV task occurred only in patients younger than 13.

Independently from psychophysiological alterations, patients younger than 13 showed some impairment in tests exploring visual-spatial and visual memory, sustained and shared attention. The occurrence of neurocognitive disorders in early-treated PKU has been the object of several studies over the last 10 years (46). A meta-analysis focusing on the speed of information processing in early-treated PKU, concluded that RT and choice RT were increased as function of concurrent levels of blood Phe with a threshold effect that was <320 and <570 μM in children and adolescents, respectively, thus suggesting an age-related vulnerability of immature brain to the abnormal concentrations of this amino acid (47). Attention, inhibition, and motor control were additional functions potentially impaired in early-treated PKU subjects in comparison with controls (48). No longitudinal designed study focusing on the developmental trajectory of neurocognitive skills in PKU subjects is so far available. So the consequences (if any) of such alterations on the neurocognitive outcome and adaptive competences in adult life remain to be explored.

While the low numerousness of the sample requires cautions in the interpretation of our results, the pattern of vulnerability we detected was congruous with other previous clinical observations.

In conclusion, our data showed that two independent measures of brain functioning, one exploring complex neurocognitive skills, the other the sensibility to a rapid increase of Phe of specific information processing networks, are impaired in younger but not in older PKU patients.

Such age-related vulnerability recommends a continuous dietary control in PKU patients during adolescence, and supports a pathogenetic model based on the relative vulnerability of the emerging functions to Phe suggesting a neurodevelopmental approach to the study of the neurocognitive outcome in PKU.

## Conflict of Interest Statement

The authors declare that the research was conducted in the absence of any commercial or financial relationships that could be construed as a potential conflict of interest.
